# Multiple gallstones causing ileus twenty years after cholecystectomy

**DOI:** 10.1093/jscr/rjac415

**Published:** 2022-09-28

**Authors:** Peter Petrillo, Donald Green, Susan Haag, John Kepros

**Affiliations:** HonorHealth Scottsdale Osborn Medical Center, Trauma Department, Scottsdale, Arizona, USA; HonorHealth Scottsdale Osborn Medical Center, Trauma Department, Scottsdale, Arizona, USA; HonorHealth Research Institute & HonorHealth Scottsdale Osborn Medical Center, Trauma Department, Scottsdale, Arizona, USA; HonorHealth Scottsdale Osborn Medical Center, Trauma Department, Scottsdale, Arizona, USA

## Abstract

Gallstone ileus is an important form of small bowel obstruction that occurs in less than 0.5% of patients who present with obstruction. A biliary enteric fistula that evolves in the setting of chronic cholecystitis may allow the passage of a large gallstone into the gastrointestinal tract distal to the common duct. A single stone that is sufficient in size (at least 2–2.5 cm diameter) may then create a mechanical obstruction, most often at the ileocecal valve or the terminal ileum where the intestinal lumen narrows, and where peristalsis is less robust. We present an unusual case of gallstone ileus in a patient whose obstruction was caused by not one, but seven individual gallstones, collectively restricted in the jejunum at the point of a previous anastomosis and occurring twenty years after cholecystectomy.

## INTRODUCTION

Gallstone ileus is characterized by an obstruction of the intestines caused by the settling of gallstones in the lumen of the intestines. The obstruction may present in any location in the intestinal tract; yet, it predominantly occurs in the terminal ileum potentially due to less active peristalsis [1, 2]. Due to late-stage gallstone disease, gallstones most often enter the gastrointestinal tract through a biliary-enteric fistula [1]. Gallstone ileus (GI) is considered a rare complication of gallstone that occurs in less than 0.05% of patients with bowel obstruction and in 25% of cases of non-strangulated small-bowel obstruction in those over 65 years of age [2]. GI mortality remains high, ranging from 12 to 27% partially due to high misdiagnosis rate, patient age and delayed hospital admissions [2, 3].

Gallstones are typically divided into three categories due to cholesterol content: cholesterol stones (cholesterol content >70%), mixed stones (cholesterol content 30–70%) and pigment stones (i.e. brown stones, cholesterol content <30%) [4]. Most reports reveal that stones smaller than 2.5 cm typically pass through spontaneously; thus, conservative treatment (decompression by nasogastric drainage) is conducted before a determination is made to remove the stones surgically [5].

GI more commonly occurs in the female and elderly (over 60-years-old) population but additional risk factors include a history of cholelithiasis, large stones (larger than 2 cm) and episodes of acute cholecystitis [3, 6]. A diagnosis for GI is often delayed, and it is characterized by a high mortality rate [7]. We present an unusual case of gallstone ileus in male patient over 65 whose obstruction was caused by seven gallstones, collectively restricted in the jejunum at the point of a previous anastomosis and occurring twenty years after cholecystectomy.

## CASE REPORT

A 81-year-old male with a past medical history of type II diabetes, coronary artery disease status post coronary artery bypass graft, hypertension and hyperlipidemia presents to the emergency department with a fourteen-hour history of sudden onset upper abdominal pain, which began the prior evening while taking his evening meal. The pain has been progressive, unrelenting and was associated with abdominal distention, nausea and two episodes of vomiting. He denied having had fever, chills or any recent similar episodes. He denied regular use of alcohol. Reported surgical history includes laparoscopic cholecystectomy twenty years ago, small bowel resection ten years ago and partial sigmoid colectomy six years ago.

**Figure 1:**
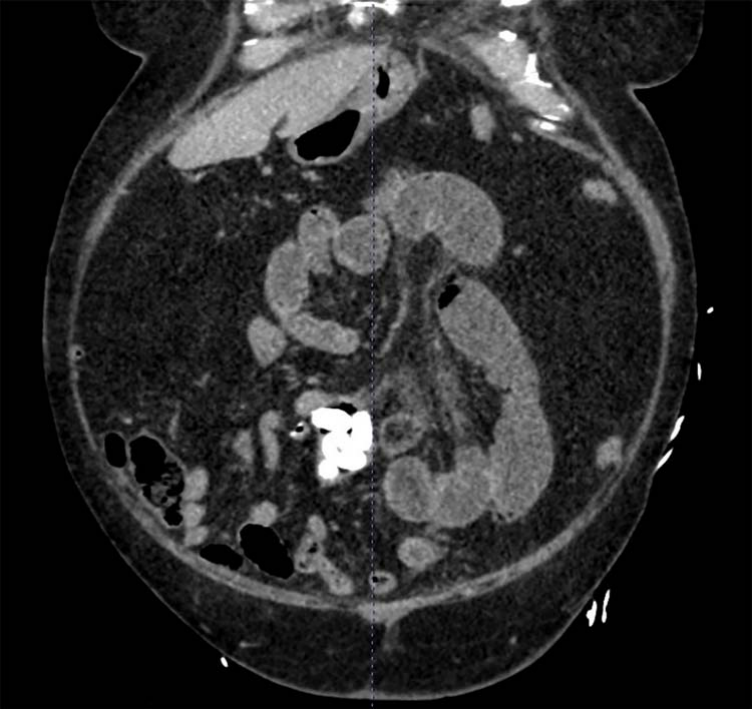
A CT scan of the abdomen and pelvis with contrast revealing a dilatation of the distal jejunum with air fluid levels and fecalization.

In the Emergency Department, his temperature was 97.2 F, HR87reg, BP 186/94, RR18, and his SpO2 98% on room air. His abdomen was diffusely tender, distended and tympanic. His WBC was 11.5, Alk Phos 67, Tbili 2.2. A CT scan of the abdomen and pelvis with contrast was obtained (Fig. 1) revealing a dilatation of the distal jejunum with air fluid levels and fecalization. Seven, oval-shaped calculi measuring approximately 1.3 cm each were distal to the area of fecalization. Immediately distal to the most distal calculus, a circumferential suture line consistent with previous small bowel anastomosis was identified. Post cholecystectomy status was verified; bile ducts were not dilated and there was no pneumobilia.

**Figure 2:**
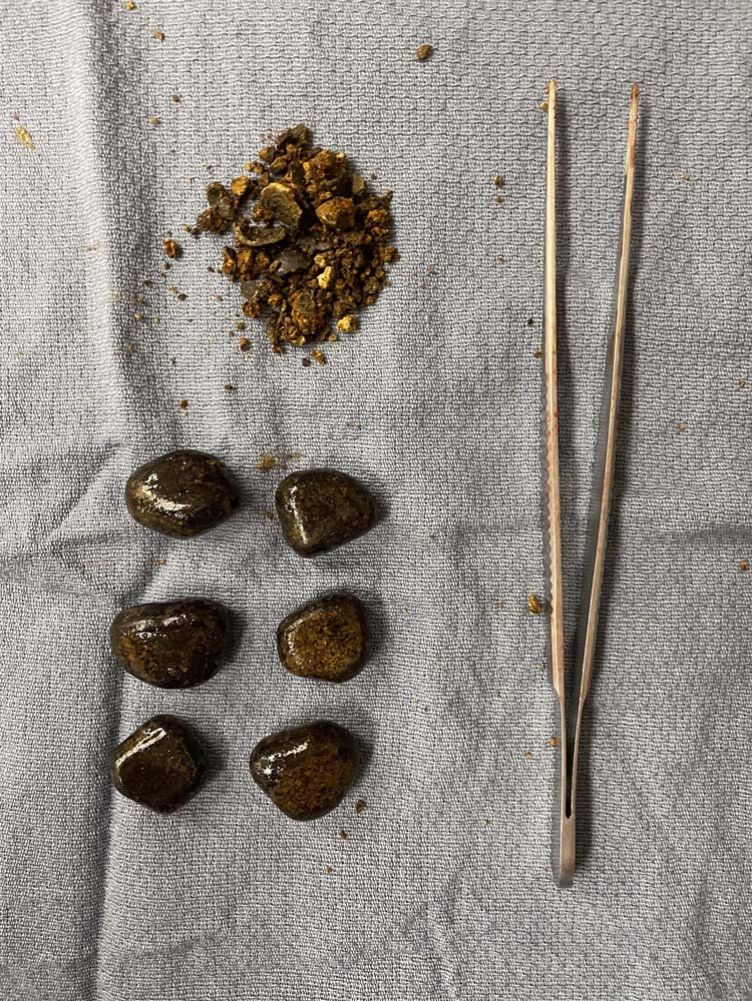
Seven large gallstones each approximately 2.0 cm in diameter were liberated.

The patient was taken to the operating room for exploratory laparotomy where the area of obstruction was readily identified. The foreign bodies were milked proximally to an area of healthy bowel and delivered through an enterotomy. Seven large gallstones each approximately 2.0 cm in diameter (Fig. 2) were liberated. The enterotomy was repaired, the peritoneal cavity was irrigated, and the fascia and skin were closed. The patient was returned to the surgical floor where he recovered bowel function, was able to take regular diet and was eventually discharged to his home in stable condition.

## DISCUSSION

Gallstone ileus is an uncommon cause of mechanical bowel obstruction, which requires a high index of suspicion to identify. Rigler’s triad of pneumobilia, ectopic gallstones and bowel obstruction is pathognomonic for the condition. The biliary enteric fistula is a complication in as many as 3% of patients with cholecystitis and is the necessary condition to allow for the direct passage of a stone large enough to cause a mechanical obstruction [8]. In the post cholecystectomy patient, the presence of gallstone ileus is rare, and it has been hypothesized that the most common presentation is developed from a spilled gallstone that subsequently erodes into the bowel. Such cases are conspicuous for their absence of pneumobilia and for the median time to presentation after cholecystectomy of six months [9]. A second theory involves the development of a residual stone within the biliary tree that may then pass into the bowel by the ampulla of vater or by fistula [9, 10]. Finally, it has been proposed that gallstones previously passed through a biliary enteric fistula are able to reside undetected within a diverticulum of the small intestine only to create trouble in the distant future [9–11].

Research shows variations in treatment approach regarding GI. Procedure selection depends on several factors, such as patient comorbidities as well as surgeon preference and technique. The typical surgical approach has been enterolithotomy. A prior study revealed that the enterolithotomy, cholecystectomy and fistula closure technique resulted in a mortality rate of 16.9% compared to 11.7% for enterolithotomy [12]. Moreover, results from one study indicated that enterolithotomy has been a better approach for the majority of patients [13].

This case is particularly interesting considering the multiples of stones, which collectively caused the mechanical obstruction at the point of a prior anastomosis of the jejunum. Indeed, swallowed foreign bodies was higher on the differential than residual gallstones given the large time interval since this patient’s cholecystectomy. No anatomical anomaly, which might have supported the occult harboring of this patient’s stones, was ever identified, and a literature review revealed no similar report of multiple stones creating obstruction after cholecystectomy. Despite post-cholecystectomy GI being rare, physicians might become more cognizant of its existence, and diagnosis may be facilitated by an abdominal CT.

## PREVIOUS PRESENTATION

Informed patient consent was obtained for this case report.
